# The aging signature: a hallmark of induced pluripotent stem cells?

**DOI:** 10.1111/acel.12182

**Published:** 2013-11-21

**Authors:** Leili Rohani, Adiv A Johnson, Antje Arnold, Alexandra Stolzing

**Affiliations:** 1 Fraunhofer Institute for Cell Therapy and ImmunologyPerlickstrasse1, 04103, Leipzig, Germany; 2 Physiological Sciences Graduate Interdisciplinary Program, University of ArizonaTucson, AZ, 85724, USA

**Keywords:** aging, differentiation, epigenetic, induced pluripotent stem, reprogramming, telomeres

## Abstract

The discovery that somatic cells can be induced into a pluripotent state by the expression of reprogramming factors has enormous potential for therapeutics and human disease modeling. With regard to aging and rejuvenation, the reprogramming process resets an aged, somatic cell to a more youthful state, elongating telomeres, rearranging the mitochondrial network, reducing oxidative stress, restoring pluripotency, and making numerous other alterations. The extent to which induced pluripotent stem cell (iPSC)s mime embryonic stem cells is controversial, however, as iPSCs have been shown to harbor an epigenetic memory characteristic of their tissue of origin which may impact their differentiation potential. Furthermore, there are contentious data regarding the extent to which telomeres are elongated, telomerase activity is reconstituted, and mitochondria are reorganized in iPSCs. Although several groups have reported that reprogramming efficiency declines with age and is inhibited by genes upregulated with age, others have successfully generated iPSCs from senescent and centenarian cells. Mixed findings have also been published regarding whether somatic cells generated from iPSCs are subject to premature senescence. Defects such as these would hinder the clinical application of iPSCs, and as such, more comprehensive testing of iPSCs and their potential aging signature should be conducted.

## Introduction

It was discovered early on that somatic cells could be reset to a pluripotent state through somatic cell nuclear transfer (Gurdon, 1962; Tada *et al*., 1997; Hochedlinger & Jaenisch, 2002; Wilmut *et al*., 2007) and cell fusion (Tada *et al*., 2001). A landmark experiment in the cell reprogramming field was performed by Takahashi and Yamanaka, demonstrating that adult somatic cells could be restored to pluripotency through the exogenous expression of four transcription factors: Oct4, Sox2, Klf4, and c-Myc. These induced pluripotent stem cells (iPSCs) expressed markers exclusive to embryonic stem cells (ESCs), mimed their morphology and growth properties, and could differentiate into all three germ layers (Takahashi & Yamanaka, 2006).

Since their initial discovery, multiple methods of reprogramming have been generated. Adult somatic cells have been successfully induced into pluripotency using viral vectors (Zhou & Freed, 2009), nonintegrating episomes (Yu *et al*., 2009), and minicircle vectors (Jia *et al*., 2010). Pluripotency can also be induced by the use of reprogramming proteins, either by direct addition of purified protein (Zhou *et al*., 2009) or with extracts from cells stably expressing reprogramming factors (Kim *et al*., 2009). More recently, effective reprogramming was achieved using synthetic mRNA (Warren *et al*., 2010), a technique our group has used to derive iPSCs from disease and healthy donors. More comprehensive listings of successfully employed methods have been reviewed elsewhere (González *et al*., 2011).

With regard to aging and age-related disease, iPSCs represent enormous therapeutic potential. Reprogramming adult, somatic cells allows for the generation of patient-specific models that have already been used to generate a wealth of information regarding disease pathogenesis, drug testing, and drug discovery (Bellin *et al*., 2012). It was previously proposed that the ability to reprogram a cell to a youthful state without affecting the differentiation program may be an effective strategy for rejuvenating an aged organism (Rando & Chang, 2012). In order for such a method to be viable, reprogramming would have to reset the aging clock, clearing the damage that accrues with age and restoring a cell to a youthful state. This would require multiple types of restoration, as somatic cells accumulate nuclear and mitochondrial mutations as well as damaged macromolecules with age. Furthermore, aging cells are characterized by distinct changes in the epigenome, telomere shortening, increased oxidative stress, and numerous other alterations (Kirkwood, 2005; Haigis & Yankner, 2010; Johnson *et al*., 2012). Such restoration is not impossible, however, as evinced by the fertilization process, where an aged sperm and egg fuse to form a zygote devoid of aging damage or any evidence of the age of the parental cells (Rando & Chang, 2012).

There are currently conflicting data regarding the ability of reprogramming to fully rejuvenate an aged somatic cell and the extent to which iPSCs mime ESCs. Moreover, contentious data exist suggesting that cells derived from iPSCs may be subject to premature senescence. This review highlights recent data relevant to these controversies and discusses the conclusions that can be currently drawn.

### Does reprogramming reset the aging clock?

#### Epigenetic memory

**Figure 1:**
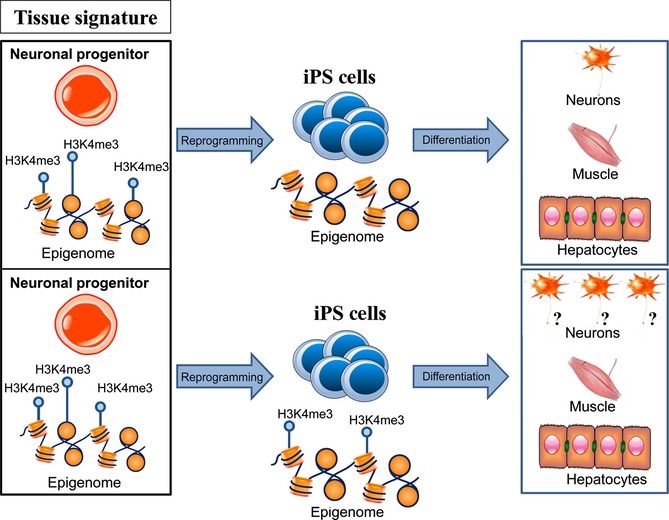
Epigenetic memory and reprogramming. There are controversial data regarding the epigenetic memory of induced pluripotent stem cell (iPSC)s and whether or not this memory affects the differentiation potential of reprogrammed cells. iPS cells have been reported to feature incomplete epigenetic reprogramming compared to ESCs, retaining residual methylation signatures characteristic of their tissue of origin that favor differentiation into lineages related to the donor cell.

Epigenetic modifications such as histone acetylation and DNA methylation play a paramount role in regulating gene expression and exhibit unique changes during aging and age-related disease (Fraga *et al*., 2007; Johnson *et al*., 2012). Modifications to epigenetic machinery can directly impact longevity (Lin *et al*., 2005) and health (Klein *et al*., 2011) as well as prevent differentiation of stem cells into somatic tissues (Bröske *et al*., 2009), highlighting the importance of a well-functioning epigenome. Emerging studies suggest that iPSCs may harbor a higher number of genetic and epigenetic abnormalities than both ESCs and the somatic cells that they originate from (Pera, 2011). Moreover, there are mixed data regarding the epigenetic memory of iPSCs and whether this memory affects the differentiation potential of reprogrammed cells (Fig. 1).

It was recently shown that low-passage iPSCs can feature incomplete epigenetic reprogramming compared to ESCs, retaining residual DNA methylation signatures that are characteristic of their tissue of origin and favor differentiation into lineages related to the donor cell (Fig. 1). iPSCs derived from mouse neural progenitors, for example, contained methylomic signatures at loci important for hematopoietic differentiation, resulting in a decreased propensity for differentiating into hematopoietic cell types. Treatment with chromatin-modifying compounds reduced DNA methylation at these loci and increased the blood-forming potential of the low-passage iPSCs, suggesting that the effects of these epigenetic marks can be attenuated via pharmaceutical intervention (Kim *et al*., 2010). Conflicting data exist regarding the retention of these methylation signatures with passage number. Some iPSC clones derived from human neonatal keratinocytes and umbilical cord blood cells were documented to maintain tissue-specific methylation memory at high passage numbers (Kim *et al*., 2011), while iPSCs derived from mouse myogenic cells, fibroblasts, and hematopoietic cells reportedly lost their epigenetic memory with continued passage in culture (Polo *et al*., 2010). More recently, genetically matched human iPSC clones from dermal fibroblasts and bone marrow stromal cells of the same donor were generated and differentiated into osteogenic and chondrogenic lineages. The authors found that, although the iPSCs exhibited an epigenetic memory characteristic of the donor tissue used, the clones varied in their differentiation propensity. Moreover, no correlation was found between the cell type of origin and the propensity of an iPSC clone to differentiate into bone and cartilage (Nasu *et al*., 2013). Further work is required to determine whether this epigenetic memory affects the pluripotency of iPSCs and whether this influence declines with passage number or varies with the donor tissue or iPSC line used.

#### Telomere length

**Figure 2:**
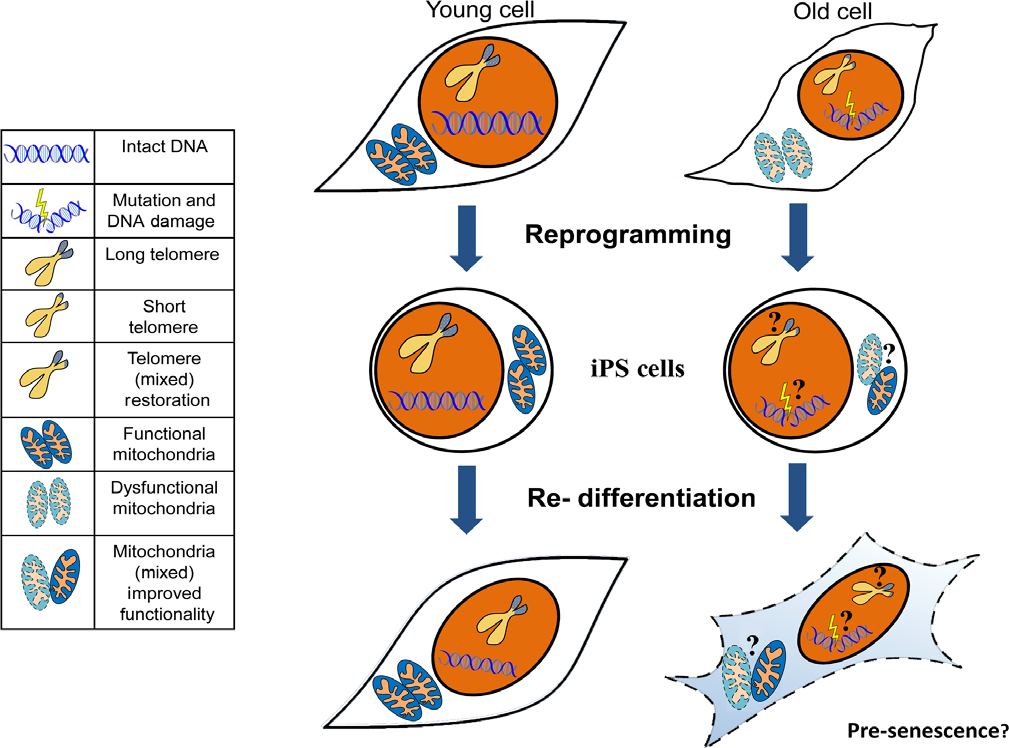
Aspects of aging and reprogramming. There are currently conflicting data regarding the ability of reprogramming to fully rejuvenate an aged somatic cell and reverse age-related changes such as DNA damage, shortened telomeres, and dysfunctional mitochondria. Moreover, contentious data exist suggesting that cells derived from induced pluripotent stem cells may be subject to premature senescence.

Unlike stem cells, somatic cells have a limited division capacity and senesce *in vitro*. The inability to further replicate is termed replicative senescence and can be induced by a plethora of factors, such as short and uncapped telomeres, oxidative stress, and mitochondrial DNA damage (Fig. 2) (Chen *et al*., 2007). The enzyme telomerase, which maintains telomere length and long-term self-renewal potential of stem cells, is strongly expressed in ESCs and is inactive in most somatic cells. As such, telomere length gradually decreases with every cell division in a typical somatic cell, eventually resulting in replicative senescence (Harley *et al*., 1990). The lifespan of normal human fibroblasts can be extended *in vitro* by exogenous introduction of plasmids expressing the catalytic subunit of telomerase hTERT, resulting in an increased telomerase activity (Bodnar *et al*., 1998). Donor cells that are difficult to reprogram can be more efficiently induced into the pluripotent state by adding hTERT and SV40 large T antigen to the reprogramming factors originally used by Yamanaka’s laboratory (Park *et al*., 2008). Furthermore, telomerase-deficient mice exhibit a sharp reduction in reprogramming efficiency that can be restored by the reintroduction of telomerase (Marion *et al*., 2009), highlighting the imperative role telomerase plays in iPS reprogramming.

Through the use of factor-based reprogramming, Yu *et al*. reported that human iPSCs display levels of telomerase activity characteristic of ESCs (Yu *et al*., 2007). In a recent report using mouse iPSCs, it was found that, at passage eight, iPSCs have shorter telomeres than ESCs, yet longer telomeres than the embryonic fibroblasts they were derived from. It was only after subsequent cell divisions and further passaging that telomeres were restored to the lengths found in ESCs (Marion *et al*., 2009). In six of seven iPS cell lines derived from human skin fibroblasts, telomeres were substantially elongated compared to their parental cells and had telomere lengths comparable to ESCs at passage five (Suhr *et al*., 2009) (Table S1). At passage 25, several iPSC clones derived from human neonatal foreskin fibroblasts had significantly longer telomeres than their parental donors (Yehezkel *et al*., 2011). iPSCs reprogrammed from senescent and centenarian human cells exhibited telomere lengths that were identical to or greater than the lengths observed in ESCs and did not shorten with increased passage number (Lapasset *et al*., 2011) (Table S1).

A study conducted by Vaziri *et al*. compared telomerase activity and telomere length in iPSCs to hESCs. The authors reported that five well-studied human iPS cell lines had significantly shorter telomeres than three commonly used ES cell lines as well as reduced levels of telomerase activity. Six iPS cell clones were then generated from the ESC-derived cell line EN13 and, after further culturing, telomeres in five of the six lines shortened to lengths comparable to those observed in the widely used iPS cell lines. One clone, however, expressed high levels of telomerase and displayed a progressive increase in telomere length for over 60 days, procuring a length equivalent to that of the parental ES cell line (Vaziri *et al*., 2010). Mathew *et al*. also reported the variability in telomere length and telomerase expression in human iPSC clones. Only clones with the highest level of telomerase expression were observed to have telomere lengths comparable to ESCs (Mathew *et al*., 2010). In iPSCs derived from patients with dyskeratosis congenita, a disease characterized by shortened telomeres and defects in telomerase function, reprogramming was capable of restoring telomere length in some iPSCs (Agarwal *et al*., 2010), but not others (Batista *et al*., 2011) (see Table S1 for a detailed overview of telomere length data). For those iPSCs where telomere length was elongated, telomere length was observed to increase with further passaging (Agarwal *et al*., 2010).

When telomere chromatin is transcribed, long noncoding RNA transcripts referred to as telomeric-repeat-containing RNA (TERRAs) are generated. TERRA levels are positively correlated with telomere length and may act as regulators of telomerase expression (Schoeftner & Blasco, 2008). In mouse iPSCs, TERRAs were observed to be increased compared to differentiated cells, yet lower compared to ESCs at passage eight (Marion *et al*., 2009). At passage 25, human iPSCs had higher TERRA levels than their parental source, yet these levels were found to vary from clone to clone (Yehezkel *et al*., 2011).

These results indicate that, although telomerase activity is clearly reconstituted during the reprogramming process, there is a significant variability in telomere length among various iPS cell lines. This length is sensitive to passage number and can even vary among cell lines derived from the same tissue or parental cell type. This is not unique, however, as it was recently shown that substantial variability in telomere length and telomerase expression exists for ESCs as well. For both iPSCs and ESCs, telomere length was found to be highly correlated with proliferation efficiency and developmental pluripotency (Huang *et al*., 2011). As such, caution should be made when drawing comparisons for telomere length, ensuring that passage number is controlled for and that appropriate controls (e.g., numerous ES cell lines and donor tissue) are used.

### Mitochondrial alterations and oxidative stress

Aging in somatic cells is accompanied by mitochondrial dysfunction and oxidative stress (Fig. 2) (Passos *et al*., 2007; Moiseeva *et al*., 2009). Compared to somatic cells, ESCs have less mitochondrial mass, decreased ATP levels, reduced reactive oxygen species (ROS), and more active repair mechanisms that mitigate mitochondrial DNA damage (Cho *et al*., 2006; Saretzki *et al*., 2008). Mitochondria in ESCs have also been reported to be sparser, have underdeveloped or condensed cristae, and rely more on anaerobic glycolysis for energy (Prigione *et al*., 2010; Suhr *et al*., 2010).

It was recently shown that the reprogramming process reorganizes mitochondria, causing them to adopt the immature morphology, distribution, and DNA content characteristic of the pluripotent state. Like ESCs, these human iPSCs also displayed reduced levels of oxidative stress, decreased amounts of intracellular ATP, and increased production of lactate. Expression of nuclear factors involved in mitochondrial biogenesis was also comparable (Prigione *et al*., 2010). Age does not appear to limit the ability of reprogramming to mediate these changes, as iPSCs with chromosomal anomalies from an 84-year-old woman exhibited restructured mitochondria and decreased levels of oxidative stress and DNA damage. These changes were similar to those observed for iPSCs generated from young donors (Prigione *et al*., 2011) (see Table S1 for details). In senescent and centenarian human fibroblasts, the reprogramming process reset the expression of genes associated with mitochondrial metabolism and oxidative stress. Mitochondrial morphology and distribution were also both comparable to ESCs (Lapasset *et al*., 2011). In iPSC clones derived from human dermal fibroblasts, mitochondrial biogenesis and ROS stress defense mechanisms were analogous to those observed in ESCs (Armstrong *et al*., 2010). Resetting of metabolic signature and restructuring of mitochondria have also been observed by others (Suhr *et al*., 2010). Varum *et al*. obtained more mixed results (Fig. 2), finding that human iPSCs exhibit a metabolic signature that, while not completely identical to ESCs, clusters more closely with ESCs than with differentiated cells. It was also observed that the mitochondrial morphology in iPSCs is more similar to ESCs than somatic cells, yet still distinct (Varum *et al*., 2011). Although different reports have been published regarding the extent of rejuvenation, these data clearly demonstrate that the reprogramming process resets mitochondria and related metabolic and stress mechanisms to a more youthful state (Fig. 2). Further studies are required to illuminate the extent of this rejuvenation, however, and why varied results have been obtained.

### Effect of donor age on reprogramming efficiency

It is currently possible to generate iPSCs from human centenarian cells (Lapasset *et al*., 2011; Yagi *et al*., 2012) that can differentiate into all three germ layers (Yagi *et al*., 2012). The reprogramming process causes senescent and centenarian cells to acquire a more youthful signature after reprogramming, resetting telomere lengths and gene expression profiles to those observed in ESCs (Lapasset *et al*., 2011). Although these data demonstrate that old age does not abrogate the ability to reprogram somatic cells, there are conflicting data on whether donor age impacts reprogramming efficiency.

Using human tissue, Boulting and colleagues (Boulting *et al*., 2011) characterized 16 iPSCs from seven individuals of different ages. The authors found that, although the lines had variable expression of early pluripotency markers, all 16 lines passed stringent tests for differentiation capacity (Boulting *et al*., 2011). In mice, dermal skin fibroblasts procured from old mice (121 weeks old) had shorter telomeres than those obtained from young mice (22 weeks old). Despite the difference in telomere length, iPSCs could be derived from both old and young donors and the resultant iPSCs had equally elongated telomeres (Marion *et al*., 2009). iPSCs generated from bone marrow of old mice (23 months old) were more difficult to produce than those generated from the bone marrow of young mice (2 months old), requiring twice as much time to reprogram (Cheng *et al*., 2011). In iPSCs sourced from 14-month-, 6-month-, and 6-week-old mice, reprogramming efficiency declined with age. Unlike those from younger mice, iPSCs from 14-month-old mice exhibited unstable pluripotency, regressing after expansion in culture and exhibiting faint staining of the pluripotency marker alkaline phosphatase. A gradual loss of expression in the ESC marker Nanog was also observed in elderly mice. This was ameliorable, however, as inhibition of BMP and TGF-ß signaling helped to stimulate self-renewal as well as to stabilize Nanog expression (Wang *et al*., 2011).

A few age-related genes have been identified that play inhibitory roles in the reprogramming process. Using mouse and human fibroblasts, Li *et al*. reported that reprogramming efficiency declined with age. Knockdown of the Ink4/Arf locus, which encodes for tumor suppressors and is upregulated with age, was sufficient to rescue the age-dependent defect in reprogramming efficiency (Li *et al*., 2009). Similarly, expression of lamin A, a protein that maintains nuclear structure integrity, increases with age in somatic cells and is expressed at lower levels in undifferentiated cells. Using human fibroblasts, an inverse correlation was observed between reprogramming efficiency and the expression level of lamin A. Like the Ink4/Arf locus, knockdown of lamin A using short hairpin RNA accelerated the rate of iPSC induction. Furthermore, overexpression of lamin A severely hindered reprogramming (Zuo *et al*., 2012). Interestingly, mutations in *lamin A/C* cause defects in the nuclear envelope and underlie Werner syndrome and Hutchinson Gilford progeria, two diseases of accelerated aging. Recently, iPSCs were generated from patients suffering from these disorders. Compared to their donor fibroblasts, these iPSCs had normal nuclear membrane morphology, suggesting that the reprogramming process could rejuvenate nuclear defects (Ho *et al*., 2011).

Although additional age comparisons are necessary, these results suggest that mammalian aging may decrease reprogramming efficiency (for an overview of donor age of the generated iPSC lines, see Table S1, and for factors used for reprogramming, see Table S2). Old age does not prevent successful reprogramming, however, as these studies demonstrate that somatic cells of any age – even those that are senescent – can be coaxed into a more youthful, pluripotent state. Moreover, the loss in efficiency can be mitigated via inhibition of specific signaling pathways and genes. Hence, old age is unlikely to nullify the rejuvenative potential of iPSCs.

### Do cells derived from iPSCs age prematurely?

Recent data have emerged suggesting that cells derived from iPSCs may exhibit signs of premature senescence (please see Fig. 2 for an overview of premature senescence in iPSCs). As with epigenetic memory and telomere length, these data are also controversial.

Suhr *et al*. reprogrammed human skin fibroblasts into iPSCs and then produced differentiated cell lines derived from three iPSC-teratoma explants. Although one line exhibited elongated telomeres, the other two displayed telomere lengths comparable to the input fibroblasts (Suhr *et al*., 2009). The same group examined the mitochondria of iPSCs generated from human fibroblasts as well as fibroblasts re-derived from iPSCs. The authors observed that the quality and function of mitochondrial complement of the re-derived fibroblasts was dramatically improved compared to the input fibroblasts (Suhr *et al*., 2010). Upon differentiation, the mitochondrial network and metabolic signature of both human ESCs and iPSCs changed to match features observed in fibroblasts. Expression of the antioxidant GPX1 was higher in fibroblasts differentiated from iPSCs, however, suggesting that iPS-derived somatic cells may differ with regard to their handling of ROS (Prigione *et al*., 2010). Feng *et al*. successfully differentiated human iPSCs into multiple cell types, although the efficiency was markedly lower than it was for ESCs. Moreover, the authors observed that, unlike cells derived from ESCs, somatic cells derived from iPSCs exhibited early senescence and possessed dramatic defects in expansion capability (Feng *et al*., 2010) (for an overview of all iPSC lines tested, see Table S1). This fate is not inexorable, however, as others have generated somatic cells from iPSCs that do not exhibit premature senescence (Gokoh *et al*., 2011).

Although it is too early to conclusively determine, issues of premature senescence may, like telomere length, vary considerably from line to line. Subsequent research drawing detailed comparisons between cell types derived from multiple ESC and iPSC lines will help resolve this contention.

## Conclusions

It is quite clear that the reprogramming reverses many aspects of aging. Even iPSCs derived from senescent and centenarian cells exhibit a more youthful signature, displaying elongated telomeres and gene expression profiles comparable to ESCs (Lapasset *et al*., 2011). Metabolic signatures, mitochondrial networks, handling of ROS, telomerase expression, and other factors are all reset to a state characteristic of pluripotency (Suhr *et al*., 2009; Prigione *et al*., 2010, 2011). These data are controversial, however, as differential reports have been published regarding the extent to which reprogramming rejuvenates aged, somatic cells and whether iPSCs exhibit aging signatures (summarized in Table S1).

Telomere length, for example, has been observed to be shortened (Vaziri *et al*., 2010), similarly sized, or even elongated compared to ESCs (Lapasset *et al*., 2011). Considerable variation in telomere length as well as telomerase activity has also been reported for both iPSCs and ESCs, however, suggesting that telomere profiles may be unique to the stem cell line used (Huang *et al*., 2011). The same may be true for epigenetic memory, as Nasu *et al*. found no correlation between the differentiation potential of an iPSC clone and the type of progenitor cell used. Instead, the authors observed that differentiation propensity varied substantially from clone to clone (Nasu *et al*., 2013). Mitochondrial networks have been reported to be rearranged to a state indistinguishable from ESCs (Prigione *et al*., 2010, 2011) or to a mixed phenotype in between that of ESCs and somatic cells (Varum *et al*., 2011). Some groups have reprogrammed cells from an elderly organism with no comment on decreased reprogramming efficiency (Marion *et al*., 2009), while others have observed reprogramming efficiency to decline with age (Li *et al*., 2009). Similar discrepancies are noted regarding premature senescence, which has been observed in somatic cells derived from some iPSCs (Feng *et al*., 2010), but not others (Gokoh *et al*., 2011).

That variations in differentiation propensity are observed in iPSCs derived from the same donor (Nasu *et al*., 2013) indicates that the reprogramming process may not reset cells to a younger state in an invariable manner. Furthermore, many of the studies cited used different reprogramming protocols as well as donor cells from different cell types or species (summarized in Table S2). Conflicting reports regarding the extent to which adult, somatic cells are rejuvenated may also be explained by the distinct protocols and materials used. Regardless, it is apparent that some iPSCs and their differentiated progeny may exhibit age-related defects. Whether these defects are cell line specific or a broader problem with iPSCs requires additional data to determine. Moreover, aging is a complex disease marked by innumerable changes, and thus far, only a small set of age-related alterations has been investigated in iPSCs. To properly assess whether iPSCs exhibit an aging signature and are less youthful than ESCs, additional research into other aspects of aging is required.

## Supporting Information

**Table S1** Aging signatures of different iPS cell lines and derivatives.

**Table S2** iPS cell lines generated from different cell types or species.
