# Supplementary material for: The aging signature: a hallmark of induced pluripotent stem cells?
Source: Aging Cell. 2013 Nov 21;13(1):2–7. doi: 10.1111/acel.12182 (PMC4326871; doi:10.1111/acel.12182)
Supplement: Supplementary file 1 — Table S1 Aging signatures of different iPS cell lines and derivatives. [file acel0013-0002-sd1.docx]

Table S1. Aging signatures of different iPS cell lines and derivatives.

| **Species** | **Donor cell type** | **Donor age** | **Aging signature^*^**  **iPS cells** | **Aging signature^*^**  **iPS-derived cells** | **Premature senescence^§^**  **iPS-derived cells** | **Reference** |
| --- | --- | --- | --- | --- | --- | --- |
| Mouse | Embryonic fibroblasts  Adult fibroblasts  Adult fibroblasts | Embryonic  Young (22 weeks)  Old (121 weeks) | (>p8): 1/2  0/1  0/1 | -  -  - | -  -  - | Marion *et al.,* 2009 |
| Mouse | Adult fibroblasts | Not mentioned | (p36): 0/1 | - | - | Huang *et al.,* 2011 |
| Mouse & Human | Mouse embryonic fibroblasts  Human adult fibroblasts  Human foreskin fibroblasts  Human fetal fibroblasts | Embryonic  Not mentioned  Young  Fetal | 0/2  1/3  1/3  0/3 | -  -  -  - | -  -  -  - | Mathew *et al.,* 2010 |
| Human | Fetal fibroblasts  Adult fibroblasts | Fetal (16 weeks)  Old (70 years) | 0/1  0/1 | 1/1  1/1 | -  - | Suhr et al., 2009 |
| Human | Adult fibroblasts | Old (70 years) | 0/5 | 4/5 | - | Suhr *et al.,* 2010 |
| Human | Foreskin fibroblasts | Young | 0/3 | 3/3 | - | Yehezkel *et al.,* 2011 |
| Human | Proliferative and senescent  adult fibroblasts  Very old adult fibroblasts | Old (74 years)  Very old (96 years) | 0/4  0/2 | 2/2  2/2 | No  No | Lapasset *et al.,* 2011 |
| Human | Fetal fibroblasts  Foreskin fibroblasts  BJ fibroblasts  FLF  ESC-derived cell line EN13 | Fetal  Young  Young  Unpublished data  Not mentioned | 1/4  1/4  1/4  1/4  2/4**^**^** | -  -  -  -  - | -  -  -  -  - | Vaziri *et al.,* 2011 |
| Human | DKC mutant fibroblasts  TERC^+/-^ mutant fibroblasts  Adult fibroblasts (wt) | Diseased  Diseased  - | 0/3**^***^**  0/3  0/2 | -  -  - | -  -  - | Agarwal *et al.,* 2010 |
| Human | TERT mutant fibroblasts  TCAB1 mutant fibroblasts  DKC1 mutant fibroblasts  Adult fibroblasts (wt**)** | Diseased  Diseased  Diseased  - | 1/3  1/3  3/4  0/3 | -  -  -  - | -  -  -  - | Batista *et al.,* 2011 |
| Human | Adult fibroblasts  AD fibroblasts  PD  fibroblasts | Very old (106 &109 years)  Diseased  Diseased | 0/1  -  - | 0/4  0/4  1/4 | -  -  - | Yagi *et al.,* 2012 |
| Human | Adult fibroblasts | DCM-diseased, aWS- diseased, and HGPS- diseased | 0/2 | 5/5 | - | Ho *et al.,* 2011 |
| Human | Foreskin fibroblasts | Young | 0/10 | 10/10 | - | Prigione *et al.,* 2010 |
| Human | Foreskin fibroblasts  Adult fibroblasts | Young  Old (84 years) | 0/7  0/7 | -  - | -  - | Prigione *et al.,* 2011 |
| Human | Adult fibroblasts | Not mentioned | 0/7 | 4/4 | - | Armstrong *et al.,* 2010 |
| Human | Foreskin fibroblasts  Fetal fibroblasts | Young  Fetal | 0/7**^#^**  0/7**^#^** | -  - | -  - | Varum *et al.,* 2011 |
| Human | Fetal fibroblasts  Foreskin fibroblasts  Adult fibroblasts | Fetal  Young  Not mentioned | -  -  - | 3/3  1/1  1/1 | Yes  Yes  Yes | Feng**^# #^***et al.,* 2010 |
| Human | Adult fibroblasts  Fetal fibroblasts | Not mentioned  Young | -  - | 0/3  0/3 | No  No | Gokoh *et al.,* 2011 |

^*^Aging signature = Tests displayed signs of cellular aging among different aging tests

^§^ This is the conclusion mentioned in the reference.

(>p8) & (p36) = Passage of iPS cells at the time of analysis

DKC = Dyskeratosis congenita

TERC = Telomerase RNA component

TERT = Telomerase reverse transcriptase

wt = wild-type

AD = Alzheimer’s disease

PD = Parkinson’s disease

DCM = Dilated cardiomyopathy

aWS = Atypical Werner syndrome

HGPS = Hutchinson Gilford progeria

**^**^**Only one clone showed a level of telomerase activity comparable to ESCs.

**^***^**DKC-mutant iPS cell line displayed telomere lengthening only with continued passage

**^#^** The iPS cell lines are not completely identical to hESCs in terms of mitochondrial morphology and metabolic gene expression.

**^# #^** Because of the very low efficiency reported for most hiPSC lines, the differentiation analysis and most of the aging analysis was done using fetal-fibroblast-derived iPS cells.
