# Supplementary material for: The aging signature: a hallmark of induced pluripotent stem cells?
Source: Aging Cell. 2013 Nov 21;13(1):2–7. doi: 10.1111/acel.12182 (PMC4326871; doi:10.1111/acel.12182)
Supplement: Supplementary file 2 — Table S2 iPS cell lines generated from different cell types or species. [file acel0013-0002-sd2.doc]

Table S2. iPS cell lines generated from different cell types or species.

Species	Name of  iPS	Factors used	Donor cells	iPS origin	ESC	Pluripotency standards	Reference	
Mouse 	3F- iPS
4F- iPS	OSK
OSKM
(retroviral)	MEF
MSF	Own (Takahashi et al 2006, Nakagawa et al 2008, Blelloch et al 2007, modified)	mESC cl1
mESC cl2
	Done (gene array & in vitro differentiation : N/A	Marion et al., 2009	
Mouse 	iPS-3
iPS-5
iPS-6	OSKM (retroviral)	adult fibroblasts	Own ( Okita K 2007)	WT Terc+/+mESC,
Het Terc+/–mESC,
G1,G3,G4 Terc–/–mESC	Done (Methylation, Differentiation & Karyogramm : N/A)	Huang et al., 2011	
Mouse & Human	N/A


NHF5–10
NHF5–11
NHF5–38


hiPSC-FSC


hiPSC-IMR90	OSKM (retroviral)


OSKM (retroviral) & NL (lentiviral)

OSNL (lentiviral)


OSNL (lentiviral)	MEF


Human adult fibroblasts


Human foreskin fibroblasts

Human fetal fibroblasts	Own (Takahashi et al 2007)

Own (Takahashi et al 2007 & Yu et al 2007)

(Yu et al 2007)


(Yu et al 2007)	HM1 mESCs
H1, H9 hESC	Done (gene array & karyogramm: N/A)

Done (only for pluripotent markers)


Done


Done
	Mathew et al., 2010	
Human 	IPSCA1
IPSCA2 
IPSCA3
IPSCA4


IPSCB1
IPSCB2
IPSCB3	OSKM
OSNL
(lentiviral)	Fetal fibroblasts (16 weeks of gestation) 


Adult fibroblasts (70 years)	Own (Yu et al 2007 & Takahashi et al 2007)	H1, H7, H9	Done (Methylation & Karyogramm: N/A)	Suhr et al., 2009 & 2010	
Human	iPS-15
iPS-35
iPS-47	OSK 
(retroviral)	Foreskin fibroblasts	Own (Takahashi et al., 2007)	H9.1, H9.2	Done (gene array & karyogramm: N/A)	Yehezkel et al., 2011	
Human	iPSC 74P


iPSC 74S


iPSC 96	OSKMNL
(lentiviral)	Proliferative adult fibroblasts (74 years) 

Senescent
adult fibroblasts (74 years) 

very Old fibroblasts (96 years)	Own (Yu et al 2007 & Takahashi et al 2007)	H1, H9	Done	Lapasset et al., 2011	
Human	IMR90-1
iPS(IMR90)-1
iPS(IMR90)-4


iPS(foreskin)-1


BJ1-iPS1

FLF-iPS

EH1
EH2
EH3
EH6
EH6A
B2	OSKM (retroviral) & OSNL (lentiviral)

OSNL (lentiviral)

OSKM (retroviral)	Fetal fibroblasts


Foreskin fibroblasts

BJ1

Unpublished

EN13*	(Takahashi et al., 2007) & (Yu et al 2007)

(Yu et al 2007)

(Park et al 2007)
Unpublished

Own (Takahashi et al., 2007)	HI, H9, MA03	Done


Done 


Done (methylation, in vitro differentiation & teratoma: N/A)

Done	Vaziri et al., 2011	
Human 	DKC1 del37L iPS 
TERC+/- iPS 


Wild-type iPS  	OSKM (retroviral)	Adult fibroblast (from DKC and TERC+/- mutant fibroblasts)

Adult normal fibroblasts	Own (Park et al 2008)	N/A	Done (Methylation & gene array: N/A)
	Agarwal et al., 2010	
Human	TERT-iPS
TCAB1-iPS 
DKC1-iPS 


Wilde-type iPS
	OSKM (retroviral) & (lentiviral)	Adult fibroblast (from TERT, TCAB1 & DKC1 mutant fibroblasts)

Adult normal fibroblasts	Own (Byrne JA et al 2009)
Sommer CA et al 2009)	H9	Done


Done	Batista et al., 2010	
Human	iPSC clone 100–1 #8
iPSC clone 100–1 #16

iPSC clone 100–2 #1


PS1-4-iPS
PS2-2-iPS


PARK4-4 PARK4-14	OSKM (retroviral)


OSKLN
(retroviral)


OSKM (retroviral)	Adult fibroblasts (106-year-old donor)

Adult fibroblasts (109-year-old donor)

Adult fibroblasts (AD-fibroblasts)

Adult fibroblasts (PD-fibroblasts)	Own (Takahashi et al., 2007)	N/A	Done (Methylation & Karyogramm: N/A)	Yagi et al., 2012	
Human 	DCM iPS cells


aWS iPS cells


HGPS iPS cells
	OSKMNL
(lentiviral)


OSKM (retroviral)


OSKM (retroviral)	Adult fibroblasts (DCM-fibroblasts) 

Adult fibroblasts (aWS-fibroblasts)

Adult fibroblasts (HGPS-fibroblasts)	Own (Esteban MA, et al.. 2010; Cai J, et al. 2010; Lai WH et al 2010)	H1, H9	Done (Gene-Array & in vitro differentiation: N/A)	Ho et al., 2011	
Human 	iPS2
iPS4
iB4
iB5

OiPS3
OiPS6
OiPS8
OiPS16	OSKM (retroviral)	HFF1
BJ


Adult fibroblasts (84 years)	Own (Takahashi et al., 2007)	H1, H9	Done (Methylation: N/A)	Prigione et al., 2010 & 2011	
Human	hiPSC clone 1
hiPSC clone 4	OSKMN
(retroviral)	Adult fibroblasts	Own (Lowry et al 2008)	H9	Done (Methylation: N/A)	Armstrong et al., 2010	
Human	HFF1 iPS

IMR-90 iPS


AE iPS	OSNL (lentiviral)	HFF1

IMR90 (human fetal fibroblasts)
N/A	WiCell Research Institute	WA07
WA09
WA01	Don (Methylation: N/A)
	Varum et al., 2011	
Human	iPS(IMR90)-1


iPS(Foreskin)1-1
iPS(Foreskin)4-1
iPS(Foreskin)4-3

rv-hiPS01-04	OSNL (lentiviral)

OSNL (lentiviral)

OSKM (retroviral)	Fetal fibroblasts

Foreskin fibroblasts

Adult fibroblasts	 (Yu et al. 2007)

(Yu et al. 2007)

(Takahashi et al. 2007)	H1, H7, H9	Don (Methylation: N/A)

Don (Methylation: N/A)

Done 
	Feng et al., 2010	
Human	253G1 
253G4

201B7 
201B2

#25
	OSK (retroviral)

OSK M (retroviral)

OSK M (retroviral)	Adult fibroblasts

Adult fibroblasts

Fetal fibroblasts
	(Nakagawa et al., 2008)

(Takahashi et al., 2007)

National
Research Institute for Child Health and Development	KhES-1, KhES-3	Done (methylation & gene-array: N/A)

Done


N/A	Gokoh et al.,  2011	


3F- iPS = three factors-iPS (Oct3/4, Sox2, and Klf4)
4F iPS = four factors-iPS (Oct3/4, Sox2, Klf4, and cMyc)
MEF= mouse embryonic fibroblasts
MSF = murine skin fibroblasts
WT Terc+/+ = wild-type Terc+/+ mice 
Het Terc+/– = heterozygous Terc+/– mice
G1, G3, G4 Terc–/– = early to late Terc–/– mice
DKC = Dyskeratosis congenital
TERC/ TERT = telomerase RNA component
AD-fibroblasts = fibroblasts from patient with Alzheimer Disease
PD-fibroblasts = fibroblasts from patient with Parkinson Disease
DCM-fibroblasts = fibroblasts from patient with dilated cardiomyopathy and conduction system defect
aWS-fibroblasts = fibroblasts from patient with atypical Werner syndrome
HGPS-fibroblasts = fibroblasts from patient with Hutchinson Gilford progeria
HFF = human foreskin fibroblasts
*EN13 = ESC-derived cell line
